# An ensemble learning approach to digital corona virus preliminary screening from cough sounds

**DOI:** 10.1038/s41598-021-95042-2

**Published:** 2021-07-28

**Authors:** Emad A. Mohammed, Mohammad Keyhani, Amir Sanati-Nezhad, S. Hossein Hejazi, Behrouz H. Far

**Affiliations:** 1 Department of Electrical and Software Engineering, University of Calgary, Calgary, T2N 1N4 Canada; 2 Haskayne School of Business, University of Calgary, Calgary, T2N 1N4 Canada; 3 Department of Mechanical and Manufacturing Engineering, University of Calgary, Calgary, T2N 1N4 Canada; 4 Department of Chemical and Petroleum Engineering, University of Calgary, Calgary, T2N 1N4 Canada

**Keywords:** Population screening, Machine learning

## Abstract

This work develops a robust classifier for a COVID-19 pre-screening model from crowdsourced cough sound data. The crowdsourced cough recordings contain a variable number of coughs, with some input sound files more informative than the others. Accurate detection of COVID-19 from the sound datasets requires overcoming two main challenges (i) the variable number of coughs in each recording and (ii) the low number of COVID-positive cases compared to healthy coughs in the data. We use two open datasets of crowdsourced cough recordings and segment each cough recording into non-overlapping coughs. The segmentation enriches the original data without oversampling by splitting the original cough sound files into non-overlapping segments. Splitting the sound files enables us to increase the samples of the minority class (COVID-19) without changing the feature distribution of the COVID-19 samples resulted from applying oversampling techniques. Each cough sound segment is transformed into six image representations for further analyses. We conduct extensive experiments with shallow machine learning, Convolutional Neural Network (CNN), and pre-trained CNN models. The results of our models were compared to other recently published papers that apply machine learning to cough sound data for COVID-19 detection. Our method demonstrated a high performance using an ensemble model on the testing dataset with area under receiver operating characteristics curve = 0.77, precision = 0.80, recall = 0.71, F1 measure = 0.75, and Kappa = 0.53. The results show an improvement in the prediction accuracy of our COVID-19 pre-screening model compared to the other models.

From what we know about COVID-19, more than 40% of infected people show no to very moderate symptoms, significantly contributing to the disease's non-intentional spread^1^. This situation mandates prompt and precise identification of COVID-19 through frequent and widespread testing to prevent community outbreaks. The world health organization (WHO) has identified and updated several symptoms of COVID-19, such as high temperature, coughing, and breathing difficulties^2^. However, these symptoms are common for several respiratory diseases and not necessarily unique to COVID-19, rendering it difficult for patients to self-assess. The gold-standard method for diagnosing COVID-19 uses reverse transcription-polymerase chain reaction (rRT-PCR) in nasopharyngeal (NP) swabs. However, sample collection with the NP swab is an invasive method and is not ideal for screening, prognostics, and longitudinal monitoring purposes, given that it requires close contact between healthcare providers and patients. This contact introduces a significant risk of viral transmission to healthcare providers and other patients and burdens healthcare systems. The longitudinal monitoring and early pre-screening of individuals suspicious of COVID-19 could be improved substantially with new non-invasive and easy-to-implement approaches that can be carried out efficiently at a low-cost by patients themselves without professional help.

Given the difficulties and bottlenecks experienced so far around the world with the implementation of widespread testing, the ideal test procedure would, while maintaining a high level of accuracy (sensitivity and specificity), (a) allow patients to self-assess without the need for physical contact with healthcare professionals, (b) bring down the cost per test substantially (ideally close to zero), (c) eliminate the dependency of diagnostic kits on scarce materials, manufacturing capacity, and supply chain bottlenecks, and (d) be rapidly deployable around the world without dependency on logistics and physical distribution bottlenecks. In this work, we pursue a digital method of COVID-19 testing based on audio recordings that would satisfy all four criteria. It is noted that the cough-based COVID-19 testing does not aim primarily to replace gold-standard diagnostic methods but to be used as a longitudinal and pre-screening approach for better management of COVID-19.

Computer representations of sound data may reveal information that is not detectable by humans. There is also mounting evidence that machine learning methods can detect COVID-19 signatures in cough sounds^3–6^. Since cough sounds can easily be converted to information signals and stored as digital files, and they may allow us to create a purely digital COVID-19 testing and offer enormous economic benefits. In essence, a purely digital COVID-19 testing is an 'information good' that benefits from the many favourable economics of information goods, such as zero marginal cost of production and distribution^7,8^. If the user can employ the digital test without professional supervision, it becomes instantly usable anywhere, anytime, and by anyone. Therefore, the benefits of a digital COVID-19 test are significant enough to merit its pursuit.

Several studies have collected and analyzed cough sound data for COVID-19 pre-screening testing using mobile devices and Web technology^9–12^. If enough data is available, artificial intelligence (AI) techniques would be leveraged to design and deploy COVID-19 detection models. However, publicly available datasets of cough sounds containing a substantial number of COVID-19 positive cases are limited, so data availability represents a bottleneck for training such machine learning models. The work illustrated in Imran et al.^10^ provided a proof-of-concept COVID-19 pre-screening model. They used smartphones to collect cough recordings from a limited number of COVID-positive patients (70 patients) as well as cough recordings from healthy people (247 samples) and patients with other pathological diseases (96 bronchitis and 130 pertussis patients). The coughs were then used to train three machine learning models to detect coughs of COVID-positive patients. The data and trained models for this study are not publicly available.

In another study, a crowdsourced database that contained more than 10,000 cough samples (at the time of writing) was collected from 7000 unique users, 235 of whom self-declared to have been diagnosed with COVID-19^9^. Neither the dataset nor the models developed are publicly available. A publicly and verified crowdsourced COVID-19 cough dataset was presented by Sharma et al.^11^. Although the dataset contains more than 14,000 unique subjects, less than 10% are identified as COVID-19 positive. Such highly imbalanced datasets have limitations to be used for training machine learning models for pre-screening the patients. Another dataset of cough sounds collected from media interviews of COVID-19 patients was presented, known as NoCoCoDa^12^. This database has cough sounds from only ten unique subjects, which is very limited to train machine learning algorithms. Another publicly available dataset of 121 segmented cough samples was collected from 16 patients^4^. The data also contains clinical annotation, which is accurate given its collection at a hospital under supervision. The cough samples were pre-processed and labelled with COVID-19 status acquired from polymerase chain reaction (PCR) testing, along with patient demographics (age, gender, medical history). More details on the datasets presented above can be found in the “Materials and methods” section^4,11^.

Coughing is a common symptom for over one hundred pathological conditions^13^. Support Vector Machine (SVM), Neural Network (NN), and K-Nearest Neighbour (KNN) algorithms have been utilized to analyze cough and breath sound recorded using smart-phones^14,15^ for different diseases, such as chronic obstructive pulmonary disease (COPD)^16^, tuberculosis^17^, and respiratory disorders like asthma and pneumonia. Nonnegative Matrix Factorization (NMF), SVM, CNN, logistic regression algorithms were used to extract features to analyze speech and cough sounds^18–24^. The features included the number of peaks in the energy envelope of the cough signal and the power ratio of the two frequency bands of the second phase of the cough signal. The results showed that these features could classify dry and wet coughs, enabling the identification of associated diseases. Mobile applications and Web services were developed for COVID-19 cough sound data collection and pre-screening^25–28^. However, none of the datasets are publicly available for replication.

Bagad et al.^3^ collected a large dataset of microbiologically confirmed COVID-19 cough sound from 3621 individuals, of which 2001 had tested positive. While voice and breathing sounds were collected and manually verified, only the cough sounds were used for model training. They applied a CNN model and showed a statistically significant signal predicting the COVID-19 status with the area under the receiver operating characteristic curve (AUC = 0.72). Due to the imbalanced nature of the collected dataset (more negative vs. positive cases), the authors performed two data augmentations to enrich the minority class (COVID-positive) by adding external background environment sounds from the ESC-50 dataset^29^ creating different time and frequency masking of the input spectrogram^30^. Furthermore, the authors randomly sampled 2-s overlapped segments from the entire cough segment and used short-term magnitude spectrograms as input to the CNN model.

A study conducted by MIT researchers claimed that COVID-19 patients, specifically asymptomatic patients, could be accurately identified from a forced-cough cell phone recording using CNN models^31^. They collected a balanced audio COVID-19 cough dataset (not publicly available) with 5320 patients. They developed a CNN-based speech processing framework that leverages acoustic features to pre-screen COVID-19 from cough recordings. Cough recordings were transformed with Mel Frequency Cepstral Coefficient (MFCC) and trained with a CNN ensemble model. The ensembled model was composed of a CNN model trained on the Poisson transformed MFCC layer representing the patient's muscular degradation. Three parallel pre-trained ResNet50 models tuned on speech recordings representing the patient's vocal cord, sentiment, lungs and respiratory tract characteristics. The results showed that the CNN model achieved COVID-19 sensitivity of 98.5% with a specificity of 94.2% (AUC: 0.97).

Moreover, for asymptomatic patients, the trained model achieved a sensitivity of 100% with a specificity of 83.2%. The CNN model was trained on 4,256 patients and tested on 1,064 patients. Each split input cough recording was split into 6-s audio segments, padded as needed, processed with the MFCC module^32^, and implemented the ensemble model.

To achieve better automation in voice/cough feature extraction, a large-scale crowdsourced dataset of respiratory sounds was collected to aid the detection of COVID-19^9^. The authors used cough and breathing sounds to identify COVID-19 distinguished from sounds from asthma patients or healthy people. The librosa module was used as the primary audio processing library, while VGGish was used to automatically extract audio features in addition to the various handcrafted features^33^. The handcrafted and VGGish extracted features were utilized in shallow machine learning algorithms (i.e., logistic regression and support vector machine). The results showed that this model could differentiate between cough and breathing sounds of COVID-19 patients and healthy users or patients with asthma (AUC = 0.8).

There are several challenges and limitations associated with the previous studies. The main challenge is data availability and quality. Even though some datasets are publicly available, the datasets are naturally limited in COVID-positive samples compared to the negative samples. Moreover, the nature of the crowdsourced data does not guarantee any noise-free recordings. The crowdsourced cough sounds may include prolonged silence periods or significant background noise, making it challenging for any machine learning model to identify valuable patterns related to COVID-19. Previous studies have used an overlapped sliding window approach to segment the cough sound files and, consequently, enrich the data of limited COVID-positive samples. The overlapped sliding window size may significantly impact the machine learning model results as it may accumulate sound information unrelated to the cough (silence) if the window size is relatively long. If the window size is small, the machine learning model may learn repetitive patterns that might not necessarily correlate with COVID-19. The previous studies based their analysis on either the MFCC or Spectrogram of the sound files and did not explore other features or representations of the cough sound files. Moreover, the lack of fully automated feature extraction limits the ability of machine learning models to learn from diverse features that may identify COVID-19.

In this work, we utilize a crowdsourced cough dataset with diverse length, pacing, number of coughs, and stochastic background noise from publicly available data^4,11^ and segment the cough sound recordings into individual non-overlapped segments to enrich the COVID-positive records. We process each recorded cough for the first time to generate multiple representations and extract automated features per record. We then employ the generated feature library to develop and examine several shallow and deep learning models. The high-performance models are selected and further aggregated into an ensemble of classifiers to produce a robust classifier to identify COVID-19 from cough recordings. We used the kappa statistic to incorporate high-rank classifiers without favouring any of the classes^34^.

## Results and discussion

The presented work identifies COVID-19 from cough sound recordings. The main challenge faced in this work is how to utilize a crowdsourced cough dataset with diverse length, pacing, number of coughs, and stochastic background noise from publicly available COVID-19 cough sounds. We provide a practical solution that segments the cough sound recordings into individual non-overlapped segments to enrich the COVID-19 positive records. We process each recorded cough to generate multiple representations and extract automated features per record. We then employ the generated feature library to develop and examine several shallow and deep learning models.

The high-performance models are selected and aggregated into an ensemble of classifiers to produce a robust classifier and identify COVID-19 patients from their cough recordings. In addition, we used the kappa statistic to incorporate high-rank classifiers without favouring any of the classes. Finally, we show the significance of the proposed classification method by comparing the proposed method to recent related works. The proposed method outperforms compared to other complicated methods.

The methods developed so far segmented the cough sound recordings into overlapped segments of unjustified length and padded the resulted segments as needed. This type of segmentation introduced undesired frequencies and led to misleading classification results. Our method was deployed into a Web App to identify COVID-19 patients from cough sounds that signal the work's potential practical significance.

There is a legitimate need for the proposed predictive models based on shallow and deep learning, wherein these models use non-medical secondary data to identify health-related conditions such as COVID-19. These predictive models can be used in large-scale real-world settings. The results on real-world datasets are promising and motivate further investigations into secondary data analysis for identification of other health-related conditions".

Here we illustrate the shallow and deep learning experimentation results on the target cough sound data extracted from crowdsourced recordings. The goal is to identify COVID-19 patients from just one cough. Table 1 shows the accuracy (average ± standard deviation) of seven different classifiers trained on each of the six representations extracted from each cough sound segment. We also use the raw data directly as input images to train these classifiers. The results show that the shallow learning models cannot explain much of the data variance. The Random Forest (RF) classifier trained with spectrogram shows the highest accuracy of 0.67, followed by the logistic regression classifier trained with spectrogram (0.66). Tables 1, 2 and 3 highlights the top-three highest (accuracy, sensitivity, specificity, precision, and negative predictive value) features per classifier. The results also show that the essential representations of the cough sounds are the spectrogram, power spectrum, MFCC, and MelSpectrum. This ranking is based on how many times a specific representation appears in the top-three highest accuracy features per classifier list. This is mainly due to the non-overlapping window used to perform the cough sound segmentation in this study. The Chroma, RAW, and Tonal representations have no significant impact in detecting COVID-19 from cough sounds. Since the other studies have not presented multiple features as we did in this study^3,9,31^, there is no comparative information presentable in this regard. As most of the classification results are close to a random chance on average across all features of the classifiers, we do not proceed with shallow learning models in the final ensemble.

**Table 1:** Classification accuracy of several shallow machine learning models.

Feature/classifier	NB	KNN	LogitReg	RF	SGD	XGB	SVM
Chroma	0.51 ± 0.01	0.54 ± 0.03	0.55 ± 0.03	0.55 ± 0.03	0.52 ± 0.03	0.53 ± 0.03	0.54 ± 0.03
MelSpectrum	0.54 ± 0.03	0.65 ± 0.03	0.63 ± 0.03	0.63 ± 0.03	0.56 ± 0.03	0.63 ± 0.03	0.63 ± 0.04
MFCC	0.55 ± 0.04	0.65 ± 0.04	0.62 ± 0.04	0.64 ± 0.02	0.57 ± 0.04	0.63 ± 0.03	0.62 ± 0.03
PowerSpec	0.54 ± 0.03	0.64 ± 0.04	0.62 ± 0.03	0.63 ± 0.02	0.57 ± 0.04	0.62 ± 0.03	0.64 ± 0.02
RAW	0.53 ± 0.02	0.59 ± 0.03	0.59 ± 0.03	0.58 ± 0.03	0.54 ± 0.03	0.58 ± 0.03	0.59 ± 0.04
Spec	0.57 ± 0.05	0.65 ± 0.03	0.66 ± 0.03	0.67 ± 0.04	0.58 ± 0.04	0.66 ± 0.02	0.65 ± 0.02
Tonal	0.52 ± 0.02	0.54 ± 0.02	0.55 ± 0.04	0.54 ± 0.03	0.52 ± 0.03	0.53 ± 0.03	0.54 ± 0.02

**Table 2:** Classification sensitivity (Sens) and specificity (Spec) of several shallow machine learning models (training phase).

Feature/classifier	NB		KNN		LogitReg		RF		SGD		XGB		SVM	
	Sens	Spec	Sens	Spec	Sens	Spec	Sens	Spec	Sens	Spec	Sens	Spec	Sens	Spec
Chroma	0.14	0.88	0.50	0.56	0.50	0.58	0.45	0.61	0.51	0.58	0.51	0.52	0.59	0.50
MelSpectrum	0.34	0.79	0.63	0.63	0.63	0.65	0.58	0.66	0.60	0.57	0.61	0.63	0.62	0.66
MFCC	0.58	0.48	0.66	0.55	0.63	0.61	0.68	0.59	0.60	0.58	0.61	0.67	0.58	0.73
PowerSpec	0.74	0.36	0.58	0.62	0.59	0.65	0.65	0.66	0.56	0.59	0.61	0.62	0.63	0.64
RAW	0.23	0.83	0.55	0.63	0.56	0.63	0.54	0.61	0.48	0.56	0.62	0.53	0.55	0.65
Spec	0.51	0.62	0.68	0.64	0.69	0.60	0.68	0.67	0.65	0.57	0.65	0.65	0.65	0.75
Tonal	0.85	0.24	0.62	0.46	0.58	0.52	0.58	0.53	0.54	0.48	0.57	0.49	0.62	0.44

**Table 3:** Classification precision (Pre) and negative predictive value (NPV) of several shallow machine learning models (training phase).

Feature/classifier	NB		KNN		LogitReg		RF		SGD		XGB		SVM	
	Pre	NPV	Pre	NPV	Pre	NPV	Pre	NPV	Pre	NPV	Pre	NPV	Pre	NPV
Chroma	0.52	0.50	0.53	0.53	0.54	0.54	0.53	0.53	0.55	0.54	0.51	0.51	0.54	0.55
MelSpectrum	0.68	0.55	0.63	0.63	0.64	0.64	0.63	0.61	0.58	0.59	0.61	0.62	0.64	0.63
MFCC	0.55	0.64	0.60	0.62	0.62	0.62	0.63	0.65	0.59	0.59	0.61	0.63	0.68	0.64
PowerSpec	0.54	0.58	0.60	0.60	0.62	0.61	0.66	0.65	0.58	0.57	0.61	0.61	0.63	0.63
RAW	0.59	0.53	0.60	0.59	0.60	0.59	0.58	0.57	0.53	0.52	0.56	0.58	0.61	0.59
Spec	0.56	0.57	0.65	0.66	0.63	0.66	0.68	0.68	0.60	0.62	0.65	0.65	0.73	0.68
Tonal	0.53	0.63	0.53	0.55	0.55	0.55	0.56	0.56	0.51	0.51	0.57	0.53	0.53	0.54

Tables 4 and 5 show the experimentation results with the three deep learning models of CNN from scratch, the original Vgg16 model, and used Vgg16 with data augmentation. The deep learning models showed a better performance compared to the other shallow learning models. It is noted that the essential features that produce the highest accuracy and AUC are the same as the list discovered by the shallow learning models. The top four features of the kappa statistic are more than 0.2, suggesting at least a fair agreement between the observed accuracy from data and the accuracy due to the classifier decision function. This comparison justifies composing an ensemble from all the features and classifiers where the kappa statistic is more than 0.2. Here, we only compose four classifier models to obtain a more accurate classifier ensemble. The ensemble is, though, created from all the features regardless of the associated kappa values. The last rows in Tables 2 and 3 represent the performance of three deep learning models following their training using all features. The high variation in the entire feature images creates a very diverse pattern that could not be captured well enough using deep learning models (maximum AUC = 0.63).

**Table 4:** Classification performance per measure per classifier (CNN from scratch and tuned Vgg16 with data augmentation) after 100 training epochs.

Feature	Scratch CNN						Tuned Vgg16 with data augmentation					
	ACC	Prec	Recall	F1	kappa	AUC	ACC	Prec	Recall	F1	kappa	AUC
SPEC	0.68	0.67	0.71	0.69	0.37	0.68	0.76	0.72	0.85	0.78	0.52	0.76
Chroma	0.55	0.56	0.53	0.54	0.11	0.55	0.63	0.65	0.56	0.61	0.27	0.63
MFCC	0.71	0.75	0.64	0.69	0.42	0.71	0.61	0.63	0.54	0.58	0.23	0.61
MelSpectrum	0.74	0.70	0.84	0.76	0.48	0.74	0.69	0.68	0.72	0.70	0.38	0.69
PowerSPEC	0.70	0.68	0.75	0.71	0.40	0.70	0.69	0.76	0.54	0.64	0.38	0.68
RAW	0.56	0.57	0.49	0.53	0.13	0.56	0.58	0.56	0.69	0.62	0.16	0.58
Tonal	0.53	0.54	0.59	0.56	0.08	0.54	0.49	0.49	0.70	0.58	− 0.02	0.49
ALL features	0.62	0.63	0.55	0.59	0.23	0.62	0.63	0.62	0.67	0.64	0.26	0.63

**Table 5:** Classification performance per feature per classifier (original Vgg16 with data augmentation) after 100 training epochs.

Feature	Original Vgg16 with data augmentation					
	ACC	Prec	Recall	F1	kappa	AUC
SPEC	0.63	0.61	0.70	0.65	0.25	0.63
Chroma	0.56	0.58	0.44	0.50	0.13	0.56
MFCC	0.61	0.60	0.63	0.62	0.21	0.60
MelSpectrum	0.62	0.63	0.56	0.60	0.23	0.61
PowerSPEC	0.60	0.63	0.46	0.53	0.20	0.60
RAW	0.57	0.63	0.37	0.46	0.15	0.57
Tonal	0.54	0.60	0.22	0.32	0.07	0.54
All features	0.53	0.52	0.88	0.65	0.05	0.53

Tables 6, 7 and 8 show the classification performances of the classifiers for training and testing the three deep learning models. The three deep learning models were trained for 100 epochs and recorded the average accuracy and standard deviation per feature. A CNN model was designed from scratch and trained on the power spectrum feature to train the other two deep learning models. The results show the highest average accuracy of 0.84 for the CNN model, followed by the accuracy of 0.8 for the Mel spectrum, 0.77 for the spectrogram, and 0.68 for MFCC. Chroma, Tonal, and the Raw data did not show an improved performance compared to the other features, consistent with the results of shallow classifiers. Although the standard deviation of all models appeared to be relatively small, overfitting is observed for all classifiers, marked by the significant difference between the average accuracy for training and testing. The overfitting is mainly due to a relatively large number of weights and hyperparameters (compared to the input training image size) that must be estimated during training. Early stopping during the training phase is an effective method to compact overfitting. However, the stochastic gradient descent algorithm used for training a CNN model may get stuck into a local minimum when one uses the 'early stopping' as a stopping criterion to terminate the training process. Another method to create a more robust classifier with resistance to overfitting is to promote independent classifier models (with different features) or aggregate them using majority voting.

**Table 6:** Average classification performance per feature for training and testing the convolutional neural network from scratch over 100 training epochs.

Feature	Training scratch CNN		Testing scratch CNN	
	Average accuracy	Standard deviation	Average accuracy	Standard deviation
SPEC	0.77	± 0.09	0.65	± 0.06
Chroma	0.63	± 0.06	0.55	± 0.03
MFCC	0.68	± 0.07	0.64	± 0.06
MelSpectrum	0.80	± 0.11	0.67	± 0.07
PowerSPEC	0.84	± 0.13	0.67	± 0.04
RAW	0.77	± 0.15	0.57	± 0.03
Tonal	0.69	± 0.13	0.53	± 0.02
All features	0.67	± 0.07	0.60	± 0.02

**Table 7:** Average classification performance for the training and testing of the tuned Vgg16 network with data augmentation over 100 training epochs.

Feature	Training tunned Vgg16 with data augmentation		Testing tunned Vgg16 with data augmentation	
	Average accuracy	Standard deviation	Average accuracy	Standard deviation
SPEC	0.75	± 0.06	0.71	± 0.04
Chroma	0.75	± 0.10	0.60	± 0.02
MFCC	0.64	± 0.04	0.59	± 0.03
MelSpectrum	0.84	± 0.10	0.67	± 0.03
PowerSPEC	0.85	± 0.10	0.68	± 0.02
RAW	0.77	± 0.09	0.58	± 0.02
Tonal	0.72	± 0.10	0.52	± 0.02
All features	0.76	± 0.10	0.61	± 0.02

**Table 8:** Average classification performance for the training and testing of the original Vgg16 network with data augmentation over 100 training epochs.

Feature	Training original Vgg16 with data augmentation		Testing original Vgg16 with data augmentation	
	Average accuracy	Standard deviation	Average accuracy	Standard deviation
SPEC	0.60	± 0.03	0.61	± 0.04
Chroma	0.56	± 0.02	0.54	± 0.03
MFCC	0.55	± 0.02	0.56	± 0.03
MelSpectrum	0.61	± 0.03	0.59	± 0.03
PowerSPEC	0.62	± 0.02	0.63	± 0.03
RAW	0.59	± 0.03	0.54	± 0.03
Tonal	0.55	± 0.02	0.54	± 0.02
All features	0.51	± 0.01	0.52	± 0.02

Tables 9, 10, and 11 present the result of ensembling the top 4 classifiers with kappa >  = 0.2. The last row of these tables shows the performance of the ensemble models resulting from all the classifiers and all features. The CNN models trained from scratch showed the highest performance compared to other models (Precision = 0.8, Recall = 0.71, F1 = 0.75, AUC = 0.77, and kappa = 0.53).

**Table 9:** The performance of ensemble model classification for the features with kappa >  = 0.2 and all the features (ACC, Prec, Recall, F1, kappa).

Ensemble model	Features with kappa > = 0.2						All features					
	ACC	Prec	Recall	F1	kappa	AUC	ACC	Prec	Recall	F1	kappa	AUC
Scratch CNN	**0.77**	**0.80**	**0.71**	**0.75**	**0.53**	0.77	0.74	0.73	0.76	0.75	0.48	0.74
Tunned Vgg16 with data augmentation	0.76	0.82	0.66	0.73	0.52	0.76	0.71	0.70	0.76	0.73	0.43	0.71
Original Vgg16 with data Augmentation	0.63	0.66	0.53	0.59	0.26	0.63	0.62	0.66	0.49	0.57	0.24	0.62
All models ensemble	0.73	0.78	0.66	0.71	0.47	0.73	**0.71**	**0.72**	**0.68**	**0.71**	**0.43**	**0.71**

Bold values indicate best performance of the classifiers.

**Table 10:** The performance of ensemble model classification for the features with kappa >  = 0.2 and all the features (specificity and negative predictive value (NPV)).

Ensemble model	Features with kappa > = 0.2		All features	
	Spec	NPV	Spec	NPV
Scratch CNN	0.82	0.73	0.73	0.75
Tunned Vgg16 with data augmentation	0.86	0.71	0.67	0.74
Original Vgg16 with data augmentation	0.73	0.61	0.75	0.60
All models ensemble	0.81	0.70	0.74	0.70

**Table 11:** The performance of ensemble model classification per feature for all classifiers.

Feature	All three models ensemble							
	ACC	Prec	Recall	F1	Spec	NPV	kappa	AUC
SPEC	0.73	0.70	0.80	0.75	0.66	0.77	0.46	0.73
Chroma	0.63	0.67	0.50	0.57	0.75	0.60	0.25	0.63
MFCC	0.67	0.69	0.61	0.65	0.73	0.65	0.34	0.67
MelSpectrum	0.74	0.72	0.78	0.75	0.70	0.76	0.48	0.74
PowerSPEC	0.67	0.71	0.56	0.63	0.77	0.64	0.34	0.67
RAW	0.59	0.60	0.50	0.55	0.67	0.57	0.17	0.59
Tonal	0.54	0.54	0.53	0.53	0.54	0.53	0.07	0.54
All features	0.63	0.61	**0.76**	0.67	0.51	0.68	0.26	0.63

Bold value indicates best performance of the classifiers.

Table 12 provides a comparison of our results with results reported in previous studies. Previous works manipulated the classifier threshold to achieve specific sensitivity and specificity of interest^3,31^. However, we set the threshold of all classifiers at 0.5 to eliminate the bias to a specific class (COVID-19 versus non-COVID-19). Our results are closest to the study^24^, where they used log-Mel spectrogram from cough sounds to train a ResNet18 CNN model and manipulated the model threshold toward producing the sensitivity of 0.9. The study of Laguarta et al.^31^ used four ResNet 50 CNN pre-trained models trained on muscular degradation and vocal cords, where the threshold manipulation was done on MFCC features to achieve AUC = 0.97.

**Table 12:** Comparison of the model developed in this work with other related works.

Study	Data splitting	Participants	Features/representation	Classifier	ACC	Prec	Recall	AUC	Threshold	Kappa
^3^	Random samples, 2 s segments	3621	Spectrogram and log-melspectrogram from coughing sounds	ResNet18	NA	NA	0.9	0.72	Manipulated to yield 90% sensitivity	NA
^9^	Used the whole audio and chunked audio	2000	Hand-crafted and Vggish extracted features including tempo and MFCC from coughing and breath sounds	Logistic regression, gradient boosting trees, and SVM	NA	0.72	0.69	0.80	NA	NA
^31^	Split the sound files into 6 s audio splits	5320	Muscular degradation, vocal cords, sentiment, MFCC	Three pre-trained ResNet50	1	0.94	0.985	0.97	Manipulated	NA
Our method	Segment the coughing sounds into a single non-overlapping coughing sound	1502	Spectrogram, MelSpectrum, tonal, raw, MFCC, power spectrum, chroma	Ensemble of CNN classifiers	0.77	0.80	0.71	0.77	0.5	0.53

This comparison is not intended to be a head-to-head comparison because several implementation details are not available.

## Conclusion and future work

This work contributes to the crucial project of developing a purely digital COVID-19 diagnostic test by applying machine learning methods to analyze cough recordings. We developed a new technique to enrich crowdsourced cough sound samples by splitting/isolating the cough sound into non-overlapping coughs and extracting six different representations from each cough sound. It is assumed that there is a negligible information loss or frequency distortion due to the segmentation^35^ (dynamic behaviour of the cough sound such as start-stop sequence or pauses). Several shallow (traditional) and deep machine learning models were trained to detect COVID-19 status (either positive or negative) using the kappa statistic (> = 0.2) to select candidate classifiers and create an ensemble model to identify COVID-19 status with better accuracy compared to individual models. Because there is a high degree of overlap between the class features, we did not reach an accuracy above 90%. However, this unbiased classification threshold ensures the minimal dependency of the predictive model on the type and pattern of classifiers. Future work can emphasize learning the similarity and difference among class labels and avoid or minimize excessive false positive (waste or resources) or false negative (untreated COVID-19 patient) results. The design and deployment of a mobile and Web app to longitudinally collect and analyze cough sounds can further support informing subjects about the algorithm's performance for their COVID-19 pre-screening.

One of the recent developments in computational neuroscience is the utilization of the spiking neural network (SNN)^36,37^, a new neural network model based on discrete events (spikes) representation over time, rather than continuous values representation used in the convolutional neural network. SNN showed considerable success in discrete event detection such as tinnitus^37^ (i.e., medical condition causes ringing ears on uneven time interval with variable intensity). Therefore, we utilize the SNN model to identify COVID-19 vs non-COVID-19 directly from the coughing sound. Furthermore, utilizing the SNN model would help us prevent any information loss (due to quantization error) when segmenting the sound files into none overlapping segments and further converting each segment into different visual representations (i.e., images).

## Materials and methods

### COVID-19 data pre-processing

Other studies used a sliding window (2 to 6 s)^4,11,38,39^ to extract information from coughing and breathing sounds. The sliding window technique is sufficient if the dataset is noise-free. The noise may include a prolonged pause period and background noise. The sliding window may capture the dynamics of the sound signal. For instance, the sliding window technique can capture the number of coughs per 'unit time' and the time between two consecutive coughs. The dynamics of the cough sound signal may positively impact the successful detection of COVID-19 cases. However, the width of the sliding window may differ based on the quality of the cough sound. When the sliding window is relatively small, the dynamics of the cough sound may not be correctly captured, which causes misleading results. The longer the sliding window length, the less the dynamics of the cough sound are captured.

An ensemble of machine learning models implemented in this study uses crowdsourced cough recordings to identify COVID-19. We randomly and manually verified 30% of the cough sound files in both datasets as a safe-guard. Our verification test agrees with the ones done in previous studies^4,11^. Table 13 shows the datasets used in our study. The dataset contains cough sounds for 1502 participants, of whom 114 participants are SARS-CoV-2 positive. It is noted that the combined total duration of cough sounds from COVID-positive participants is about 20 min and 4 s, which is considerably short compared to the combined total duration of cough sounds from the population of controls (4 h, 30 min, and 15 s). This highly imbalanced data motivates the segmentation of the positive cough sounds into non-overlapped segments (each segment contains only one coughing sound) to enrich the minority class (COVID-19 positives). After segmentation, the total number of sound samples that are COVID-positive is 638.

**Table 13:** COVID-19 cough data sources.

GitHub URL	^38^	^39^	Total
Number of participants	16	1486	1502
Number of COVID-19 positive participants	7	107	114
Positive cough sound file (sample) per participant	1	2	NA
Number of non-COVID-19 participants	9	1379	1388
Negative cough sound file (sample) per participant	1	2	NA
Total positive cough sound duration	2 min and 48 s	17 min and 16 s	20 min and 4 s
Total negative cough sound duration	2 min and 15 s	4 h and 28 min	4 h, 30 min, and 15 s
Total positive cough samples (files) after segmentation	70	568	638
Total negative cough samples (files) after segmentation	103	8145	8248
Number of male participants	10	1123	1133
Number of female participants	6	363	369

### Experimental setup

Our main goal is to learn from multiple representations of crowdsourced cough sounds to identify COVID-19 patients. More specifically, we aim to extract and integrate multiple information signals from a single cough sound to identify COVID-positive versus negative patients with adequate accuracy in a classifier without bias toward a specific population. While ensemble learning is a standard method to integrate multiple information signals^40^ (either learning and pooling different classifiers on the same dataset or using bagging or boosting methods for ensemble learning), we focus on investigating different extracted features of a single cough sound to enhance the identification of COVID-19 status without oversampling or sliding window techniques. The research hypothesis necessitates the following requirements for a successful solution: The first requirement is to enrich the original data without oversampling by splitting the original cough sound files into non-overlapping segments. Splitting the sound files allows us to increase the sample size of the minority class (COVID-19) without changing the feature distribution resulted from applying oversampling techniques. The second requirement is to use an ensemble of classifiers that act independently on each extracted information signal and utilize the value of 0.5 as a threshold to decide on the input feature classes (COVID-19 positive vs. negative). This provides those classifiers and classifier ensembles that do not favour one class over the other. The third requirement is to implement a robust inclusion/exclusion criterion to include or exclude a classifier in an ensemble.

This study utilizes several classification evaluation metrics, including AUC, accuracy (ACC), precision, recall, harmonic mean (F1), and Kappa statistic. The Kappa statistic is used as a reliability measure^34^ (the inclusion/exclusion criterion) of each classifier to include it into an ensemble for producing a more robust classifier. The range of the Kappa statistic is (− 1,1). It is interpreted as follows: values ≤ 0 imply no agreement (i.e., the observed classification results is a random chance and not due to the expected results of a classifier decision function), 0.01–0.20 as none to a slight agreement, 0.21–0.40 as fair, 0.41– 0.60 as moderate, 0.61–0.80 as substantial, and 0.81–1.00 as almost perfect agreement (i.e., the observed classification results is in 100% agreement with the expected accuracy due to the classifier decision function).

### Analysis workflow

**Figure 1:**
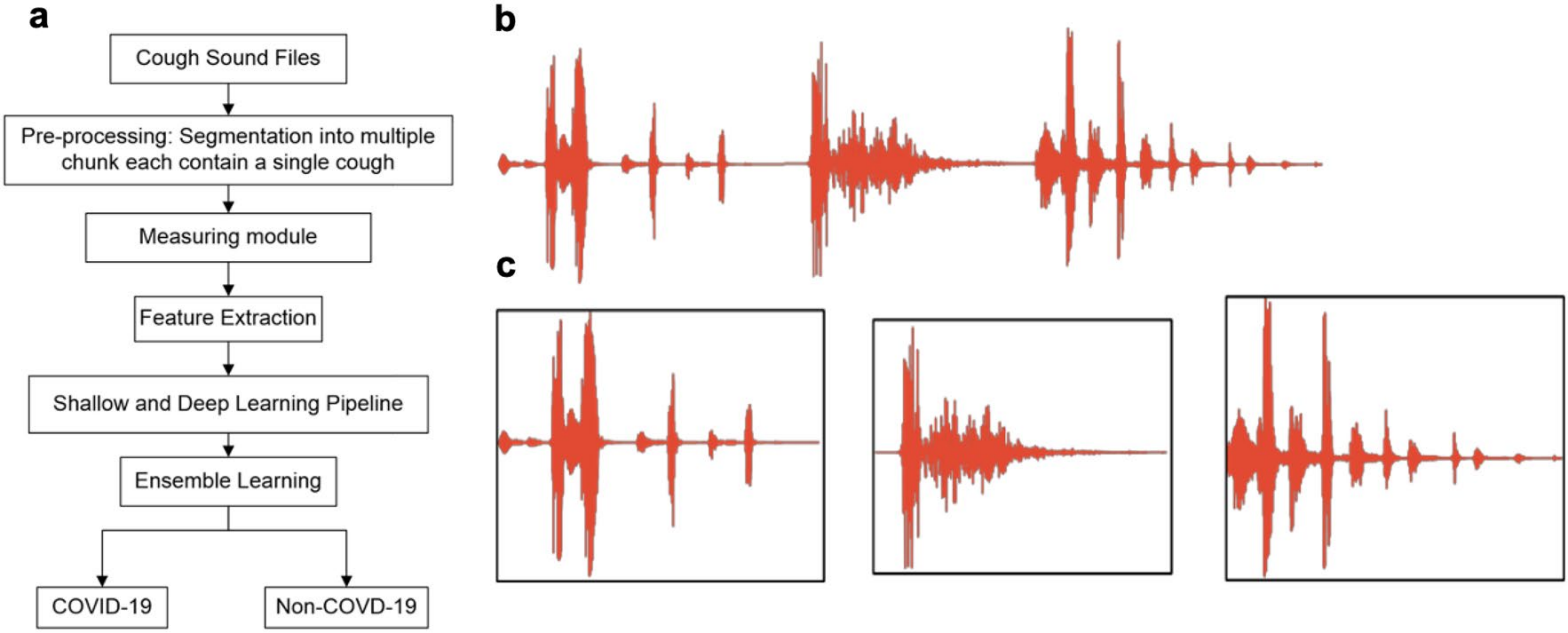
The analytical pipeline for the processing of cough sounds and the sample processed file. (**a**) The analytical pipeline for pre-processing, feature extraction, and ensemble learning from cough sounds; (**b**) Sample original cough recording sound signal; and (**c**) Segmented non-overlapped cough signals.

Figure 1a shows the analytical pipeline used in this study for pre-processing, feature extraction, and ensemble learning of COVID-19 relevant cough sounds. The pipeline starts with reading the cough audio files and segmenting them into individual non-overlapping sound files. The segmentation is conducted using the audio activity detection module to process audio files (Auditok)^41^. This module is used as a universal tool for sound data tokenization, functioning based on finding where an acoustic activity occurs in an audio stream followed by isolating the equivalent slice of the audio signal. Figure 1b shows an example of an original cough recording sound signal, and Fig. 1c shows the corresponding isolated non-overlapped signals.

**Figure 2:**
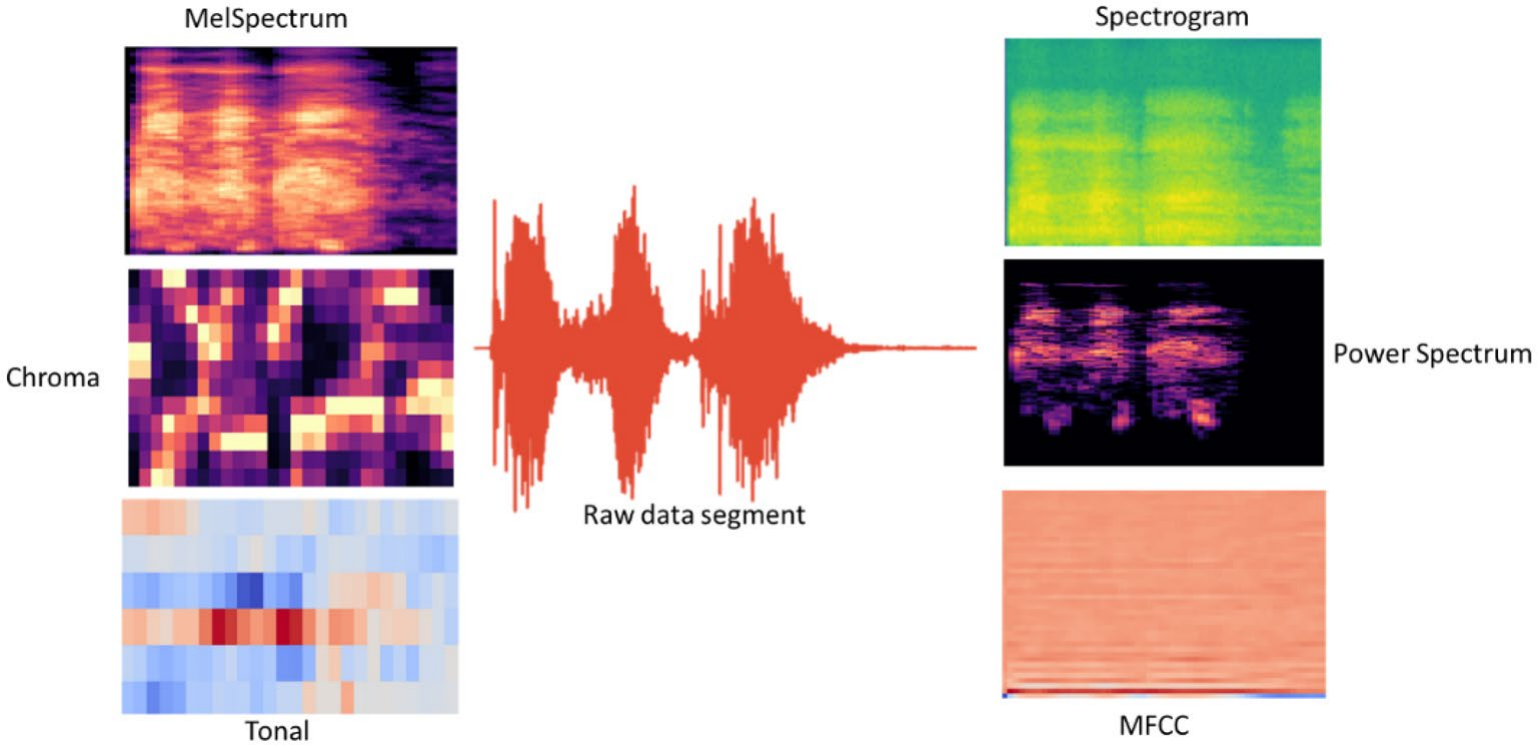
An example of raw cough sound data with the associated six representations.

Each isolated cough sound enters the measuring module following the audio splitting step to generate six different independent frequency measures (representations of the same cough sound). Each measure is converted into a reasonable resolution image (432*288 pixels) for further analysis. The Mel frequency scale is a standard audio signal representation offering a rough human frequency perception model^33^. The six measures for each isolated segment are Mel spectrum, power spectrum spectrogram, chroma, tonal, and MFCC, all based on the Mel frequency scale. Figure 2 shows an example of raw cough sound data with its associated images of the measures.

Inspired by the Vggish's model^42^ for feature extraction in audio signals, we extract features from these images using Vgg16 architecture and subject them to several shallow and deep learning models. Following the segmenting of all the positive and negative cough sound files for all participants in both datasets used in this study, we reached a total of 638 COVID-positive and 8248 negative cough sounds. We used all the 638 positive cough sounds while randomly selecting 638 negative coughing sounds to create a balanced dataset (1276 cough sound samples) for training and testing purposes. The data was divided into 80% for training (1020 images for each measure) and 20% for testing all the machine learning classifiers used in this study (256 images for each measure).

**Figure 3:**
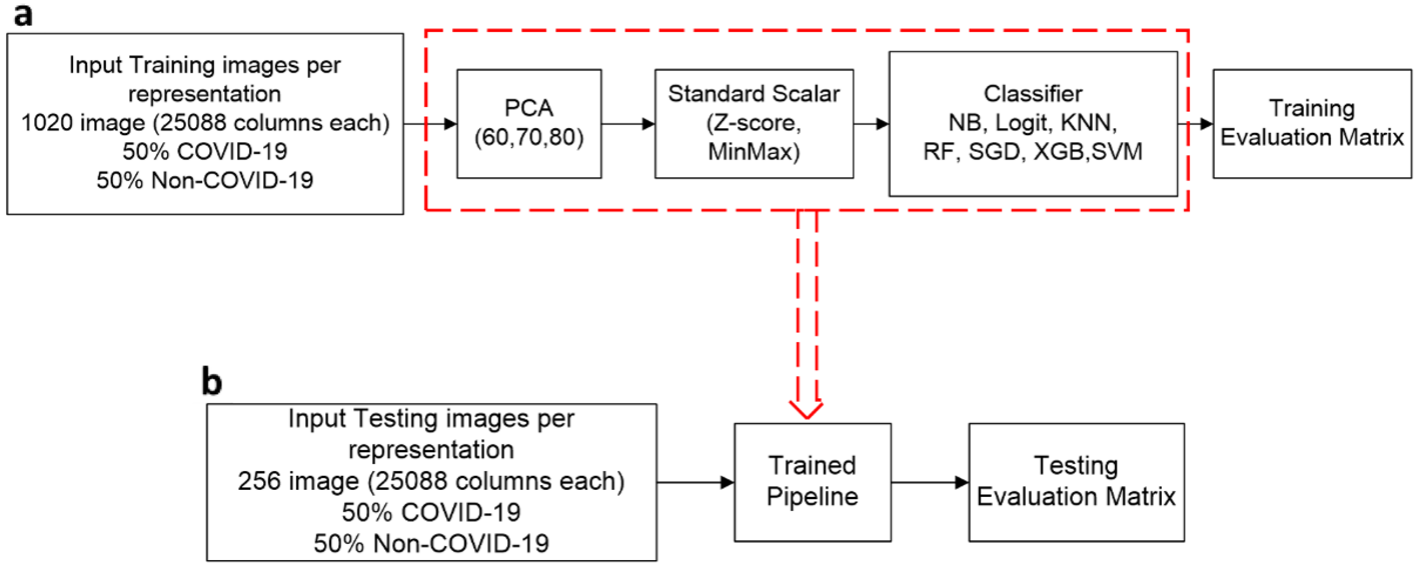
Training and testing pipelines for the shallow machine learning models used in our study. (**a**) Training pipeline: The input training images per representation are scored against the vgg19 (pre-trained model) to extract features automatically. Each representation has 1020 images in the training data, where 50% of the training data labels are COVID-positive, and the remaining 50% are negative. The features' size is 25,088, and we further select the essential features using a principal component analysis (PCA) processing step to select either 60, 70, or 80 features. The selected features are then normalized using either Z-score or MinMax standard scalars. Then the normalized features are used to train seven classifiers (Naïve Bayes (NB), Logistic Regression (Logit), K-nearest neighbours (KNN), Random Forest (RF), Stochastic Gradient Descent (SGD), Extreme Gradient Boosting (XGB), and Support Vector Machine (SVM). We measure and record the training evaluation results to choose the best classifiers. (**b**) Testing pipeline: Once the training process is completed, we score the testing data against each trained pipeline. The trained pipeline is composed of the best PCA, standard scalar, and classifier parameters. The testing data has 256 images per representation with equal labels for both COVID-positive and negative cases. We measure and record the testing evaluation results to estimate the generalization error of each pipeline.

We experiment with several traditional (shallow) machine learning models, including Naïve Bayes, logistic regression, k-nearest neighbours, random forest, stochastic gradient descent, extreme gradient boosting, and support vector machine. Figure 3 shows the overall analytical pipeline for training and testing our models. The training features are extracted using the pre-trained vgg19 model. the pre-trained model produces 25,088 feature vectors per input image. The principal component analysis was employed to reduce the dimension of the input feature and a stander scalar to normalize the input features and eventually train a set of seven classifiers. Furthermore, we experiment with three different CNN models, where one model is trained from scratch, and the other two are based on the vgg16 pre-trained model.
